# Supplementary material for: Extracellular Vesicles as Surrogates for the Regulation of the Drug Transporters ABCC2 (MRP2) and ABCG2 (BCRP)
Source: Int J Mol Sci. 2024 Apr 8;25(7):4118. doi: 10.3390/ijms25074118 (PMC11012658; doi:10.3390/ijms25074118)
Supplement: Supplementary file 1 [file ijms-25-04118-s001.zip › ijms-2910830-supplementary.pdf]

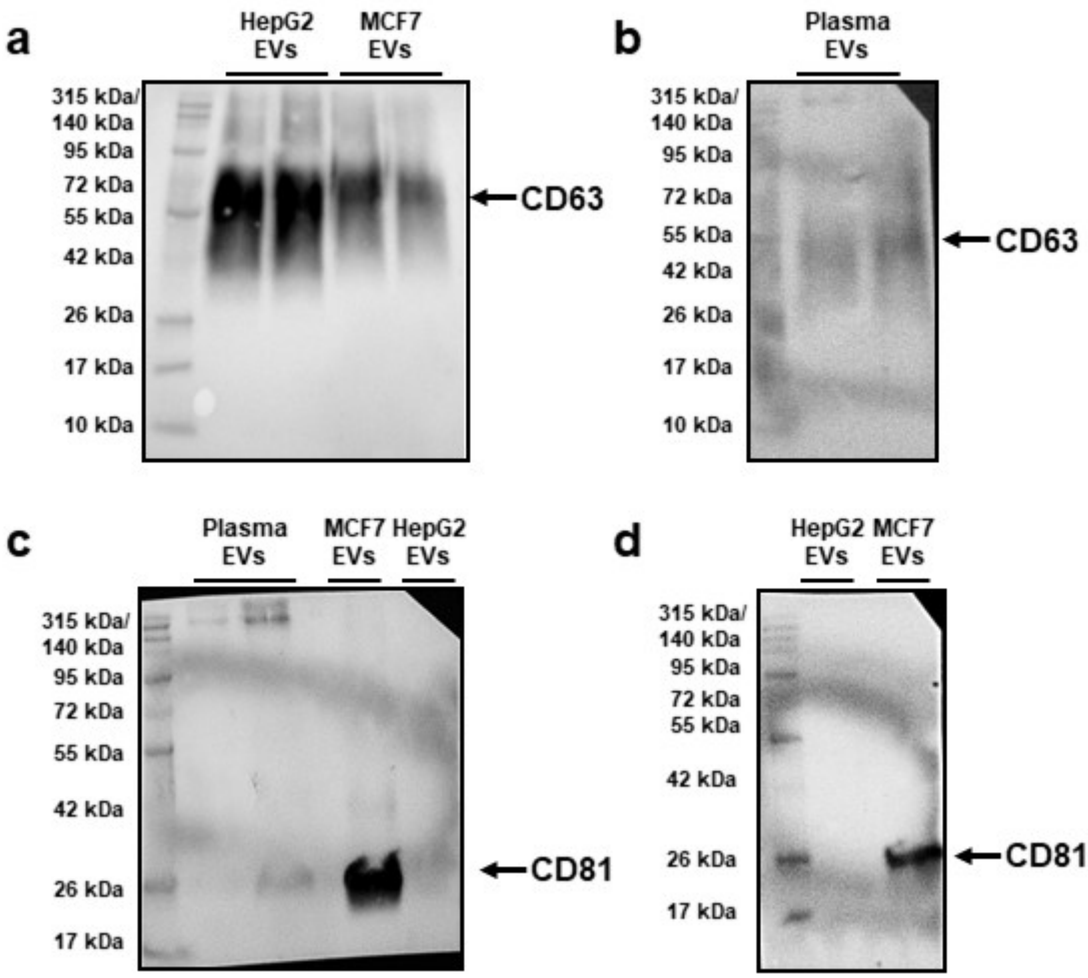

Figure S1. Western blot of the EV markers CD63 and CD81. CD63 was detected in EVs isolated from two different preparations of EVs HepG2 and MCF7 cells **(a)** and two different preparations of plasma EVs **(b)**. CD81 was analyzed in two preparations of plasma EVs, MCF7 EVs and HepG2 EVs **(c)**. CD81 was further analyzed in one further independent preparation from HepG2 and from MCF7 EVs **(d)**.

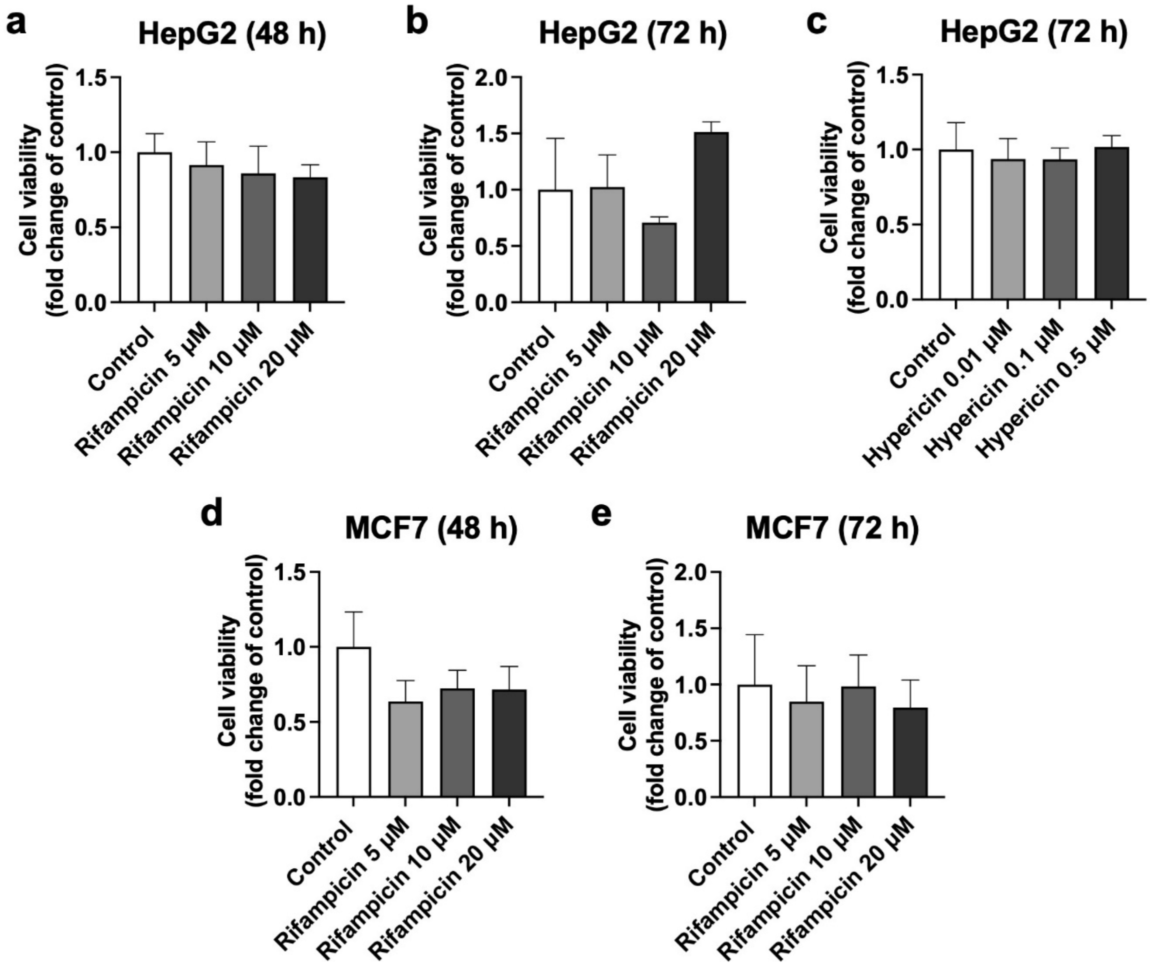

Figure S2. Effect of treatment on cell viability. HepG2 cells were treated with rifampicin (5-20 µM) for 48 (a) and 72 h (b) and with hypericin (0.01-0.5 µM) for 72 h (c). MCF7 cells were treated with rifampicin (5-20 µM) for 48 (d) and 72 h (e). Cell viability was determined by staining with crystal violet. No significant differences were observed, $n \geq 3$.

a

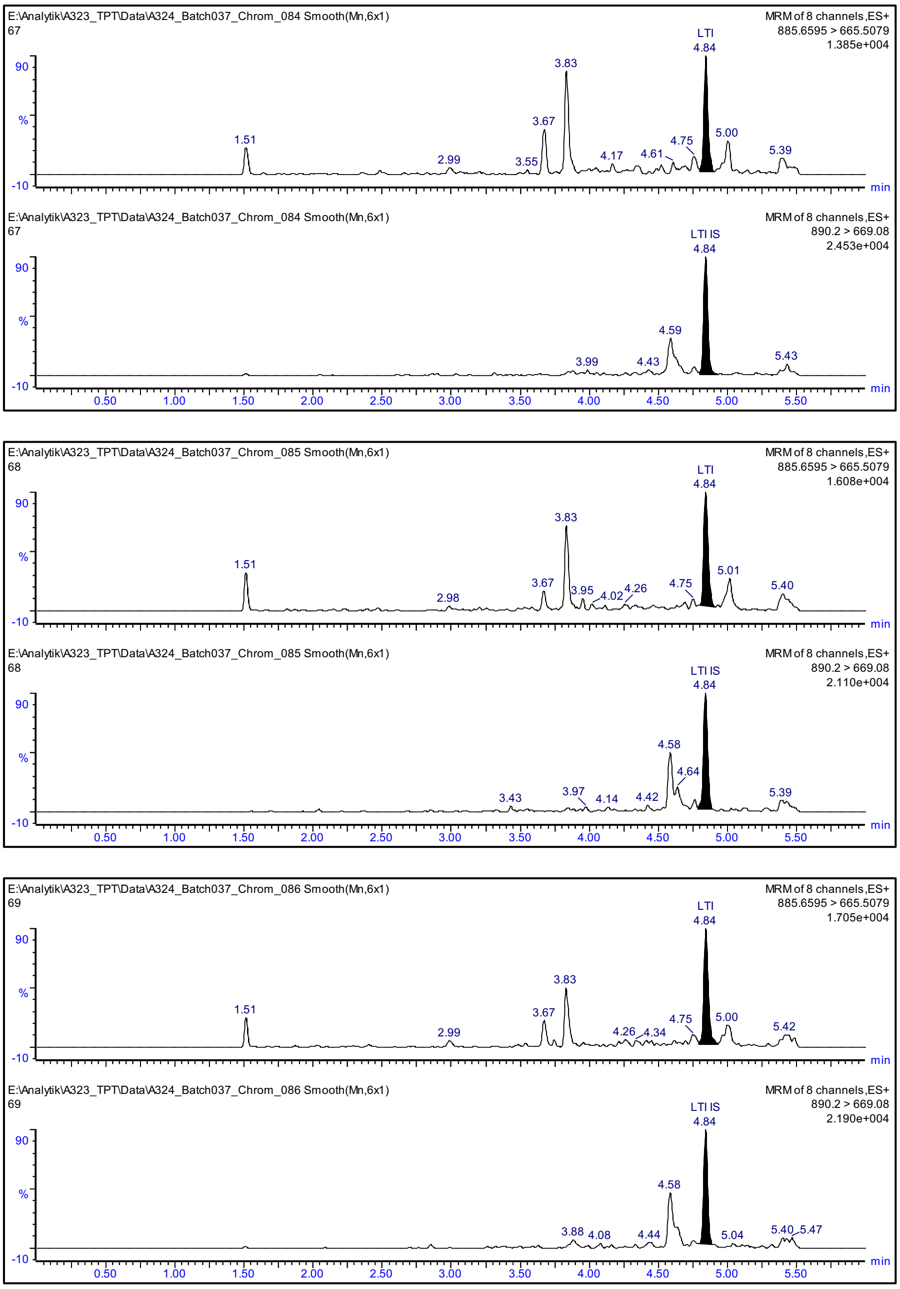
E:\Analytik\A323_TPT\Data\A324_Batch037_Chrom_084 Smooth(Mn,6x1)
67
MRM of 8 channels,ES+
885.6595 > 665.5079
1.385e+004
LTI
4.84
3.83
3.67
1.51
2.99
3.55
4.17
4.61
4.75
5.00
5.39
90
%
-10
min
E:\Analytik\A323_TPT\Data\A324_Batch037_Chrom_084 Smooth(Mn,6x1)
67
MRM of 8 channels,ES+
890.2 > 669.08
2.453e+004
LTI IS
4.84
4.59
3.99
4.43
5.43
0.50
1.00
1.50
2.00
2.50
3.00
3.50
4.00
4.50
5.00
5.50
E:\Analytik\A323_TPT\Data\A324_Batch037_Chrom_085 Smooth(Mn,6x1)
68
MRM of 8 channels,ES+
885.6595 > 665.5079
1.608e+004
LTI
4.84
3.83
1.51
5.01
2.98
3.67
3.95
4.02
4.26
4.75
5.40
E:\Analytik\A323_TPT\Data\A324_Batch037_Chrom_085 Smooth(Mn,6x1)
68
MRM of 8 channels,ES+
890.2 > 669.08
2.110e+004
LTI IS
4.84
4.58
4.64
3.43
3.97
4.14
4.42
5.39
E:\Analytik\A323_TPT\Data\A324_Batch037_Chrom_086 Smooth(Mn,6x1)
69
MRM of 8 channels,ES+
885.6595 > 665.5079
1.705e+004
LTI
4.84
3.83
1.51
3.67
2.99
4.26
4.34
4.75
5.00
5.42
E:\Analytik\A323_TPT\Data\A324_Batch037_Chrom_086 Smooth(Mn,6x1)
69
MRM of 8 channels,ES+
890.2 > 669.08
2.190e+004
LTI IS
4.84
4.58
3.88
4.08
4.44
5.04
5.40
5.47

b

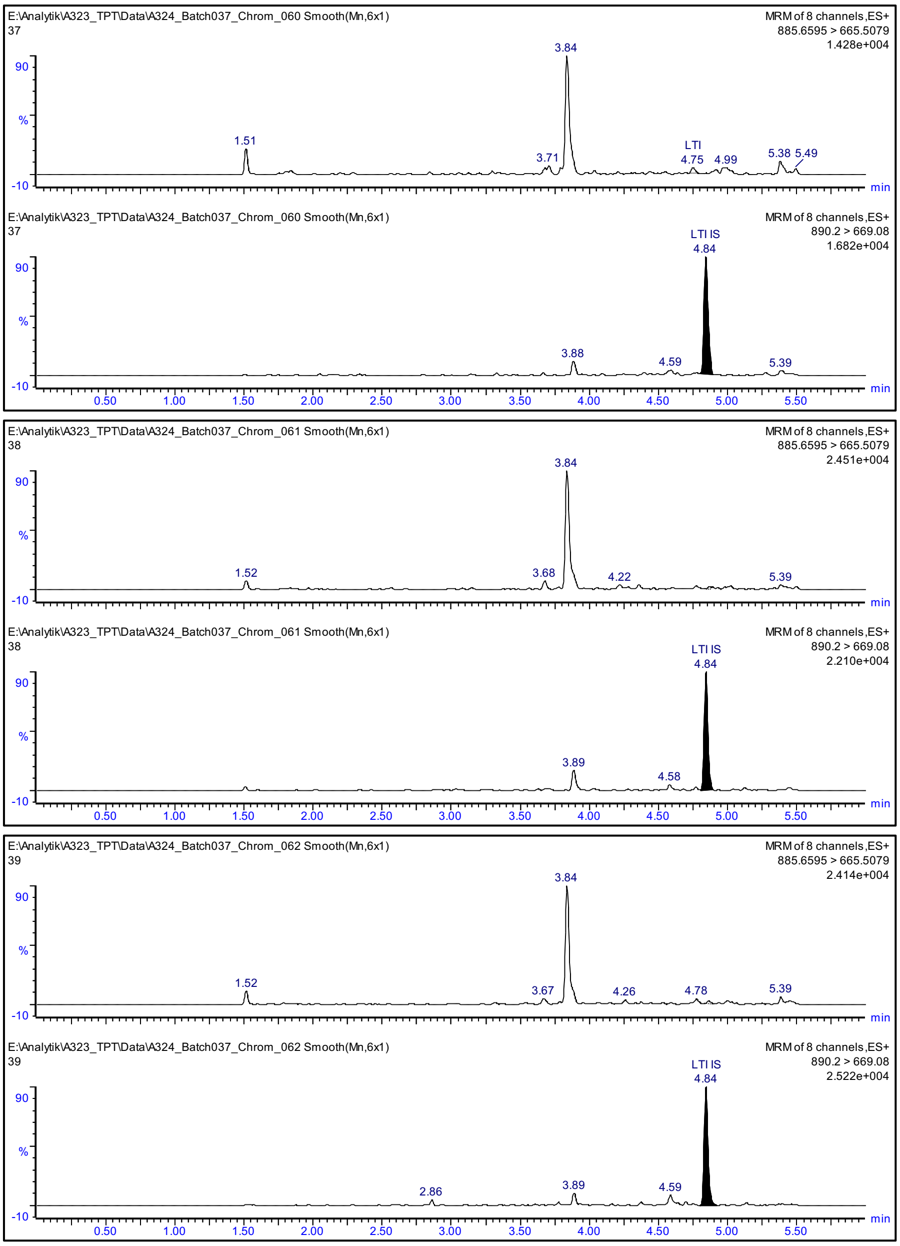

Figure S3. Comparison of ABCC2 expression in lysates from HepG2 and MCF7 cells. ABCC2 expression was determined in HepG2 **(a)** and MCF7 **(b)** cells by UPLC-MS/MS. Transporter expression was determined by quantification of the surrogate peptide LTI. All samples were spiked with a similar amount of an internal standard (LTI-IS). Three independent samples were measured. For each sample, the upper chromatogram corresponds to the LTI peptide and the lower chromatogram corresponds to the LTI-IS peptide.

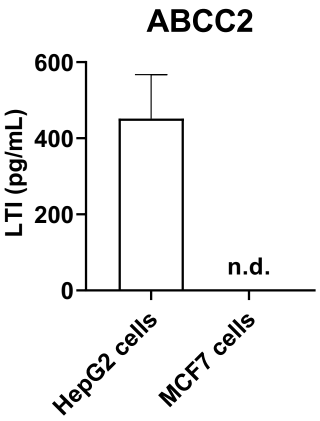

Figure S4. Comparison of ABCC2 expression in lysates from HepG2 and MCF7 cells. Expression levels (mean ± SD) were determined by UPLC-MS/MS and are presented as pg/mL of the surrogate peptide LTI. n.d.: not detected, n = 3.

a

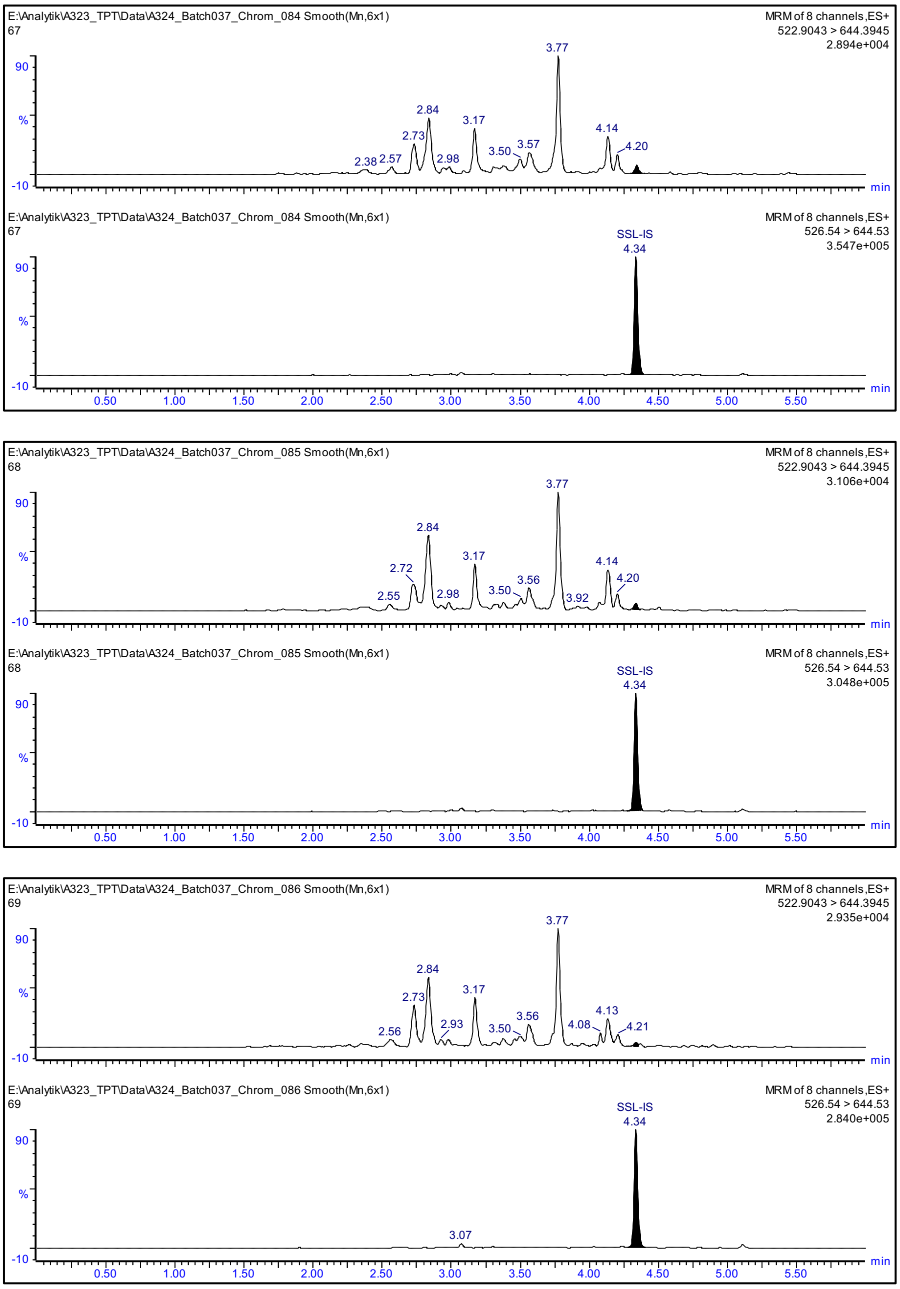

E:\Analytik\A323_TPT\Data\A324_Batch037_Chrom_084 Smooth(Mn,6x1)
67
MRM of 8 channels,ES+
522.9043 > 644.3945
2.894e+004
3.77
2.84
3.17
4.14
2.73
3.57
4.20
3.50
2.38
2.57
2.98
526.54 > 644.53
3.547e+005
SSL-IS
4.34
90
%
-10
min
0.50
1.00
1.50
2.00
2.50
3.00
3.50
4.00
4.50
5.00
5.50
E:\Analytik\A323_TPT\Data\A324_Batch037_Chrom_085 Smooth(Mn,6x1)
68
3.106e+004
2.72
3.56
2.55
3.92
3.048e+005
E:\Analytik\A323_TPT\Data\A324_Batch037_Chrom_086 Smooth(Mn,6x1)
69
2.935e+004
2.56
2.93
4.08
4.13
4.21
2.840e+005
3.07

b

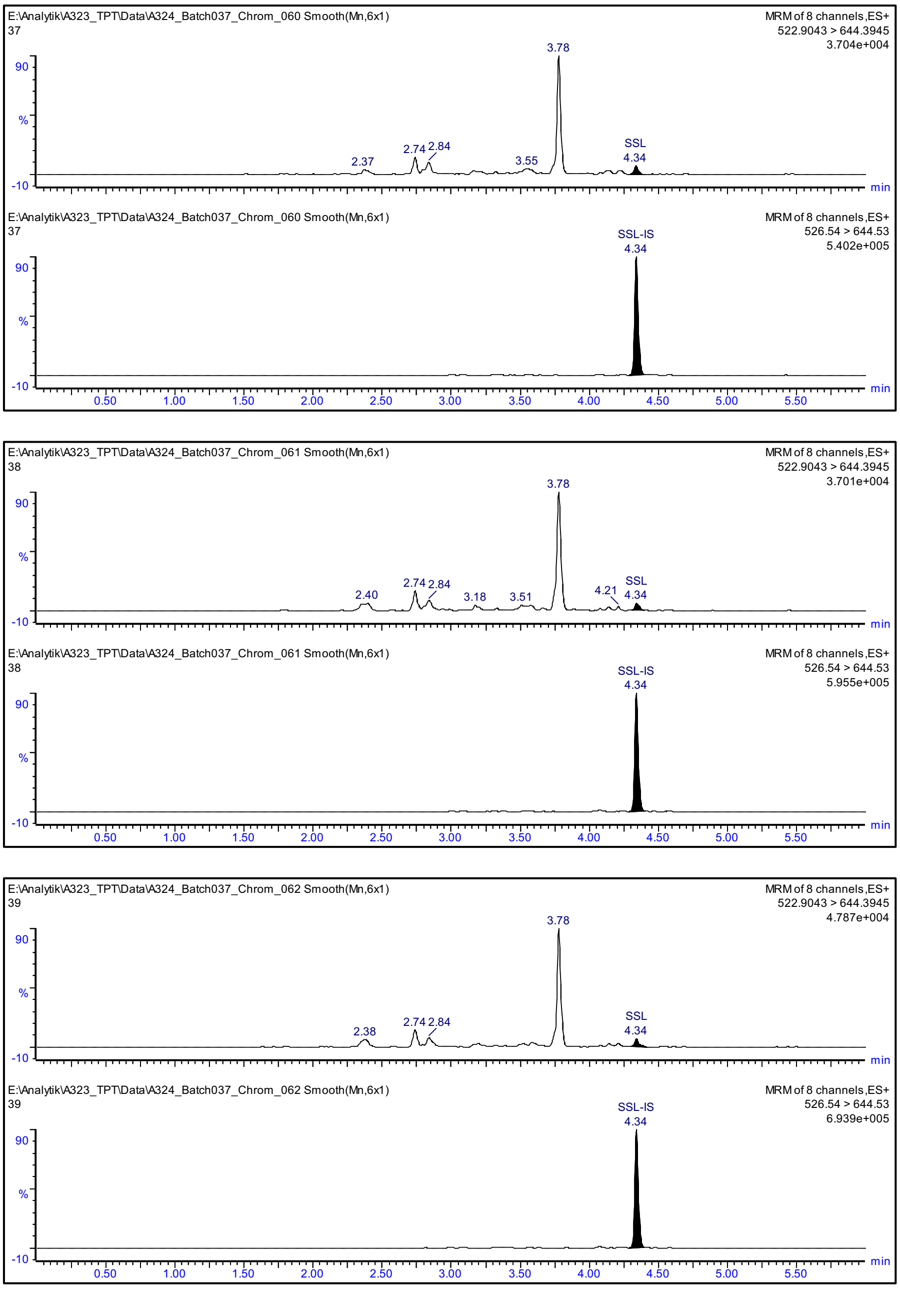

Figure S5. Comparison of ABCG2 expression in lysates from HepG2 and MCF7 cells. ABCG2 expression was determined in HepG2 **(a)** and MCF7 **(b)** cells by UPLC-MS/MS. Transporter expression was determined by quantification of the surrogate peptide SSL. All samples were spiked with a similar amount of an internal standard (SSL-IS). Three independent samples were measured. For each sample, the upper chromatogram corresponds to the SSL peptide and the lower chromatogram corresponds to the SSL-IS peptide.

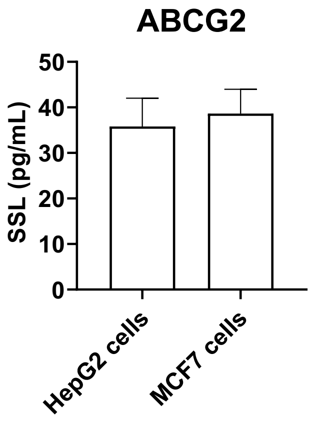

Figure S6. Comparison of ABCG2 expression in lysates from HepG2 and MCF7 cells. Expression levels (mean ± SD) were determined by UPLC-MS/MS and are presented as pg/mL of the surrogate peptide SSL. n = 3.

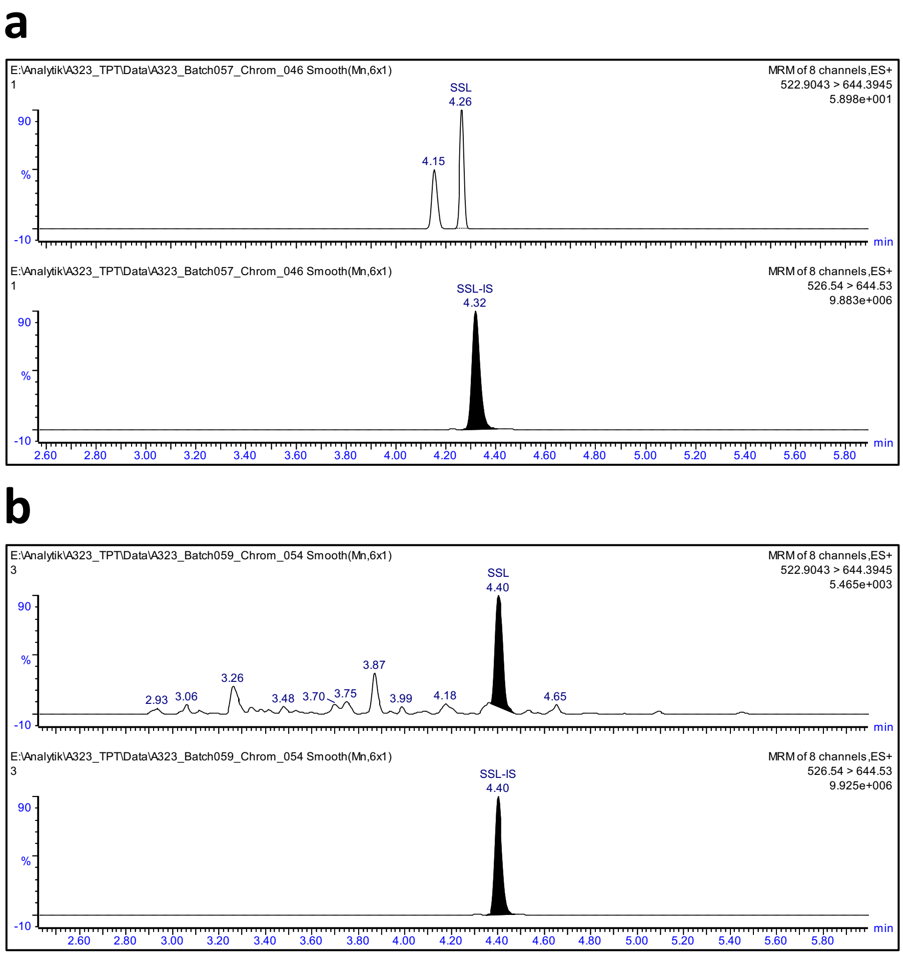

Figure S7. Comparison of ABCG2 expression in EVs from HepG2 and MCF7 cells. ABCG2 expression was determined in HepG2 **(a)** and MCF7 **(b)** EVs by UPLC-MS/MS. Transporter expression was determined by quantification of the surrogate peptide SSL. All samples were spiked with a similar amount of an internal standard (SSL-IS). Chromatograms from a representative sample are shown for each EV type. The upper chromatogram corresponds to the SSL peptide and the lower chromatogram corresponds to the SSL-IS peptide.

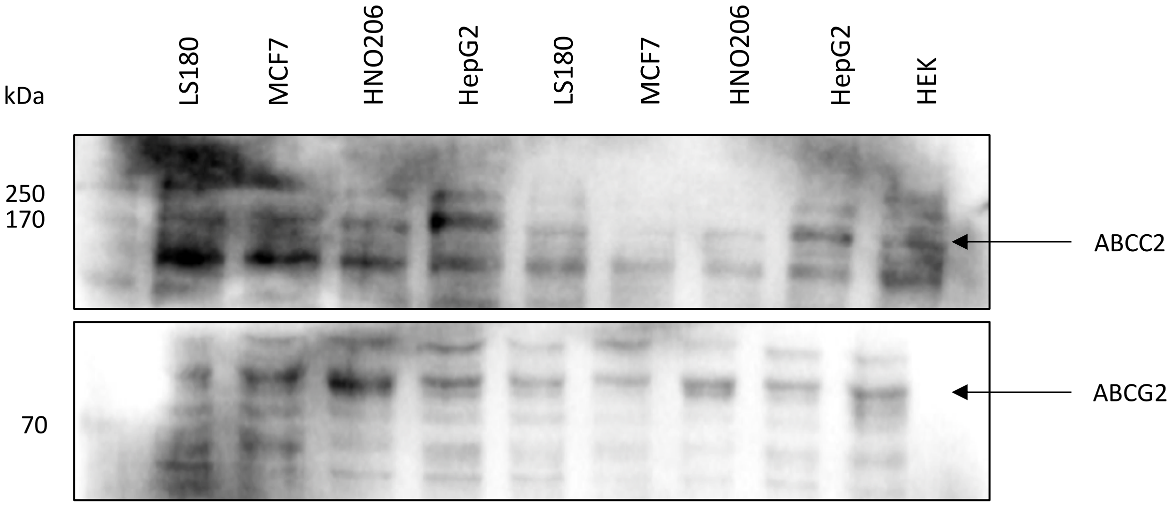

Figure S8. Comparison of ABCC2 (upper panel) and ABCG2 (lower panel) expression in lysates from different cell lines by western blot. Lysates from LS180 (colon adenocarcinoma), MCF7 (breast cancer), HNO206 (head and neck squamous cell carcinoma), HepG2 (hepatocellular carcinoma) and HEK (human embryonic kidney) were analyzed in two different preparations by western blot. Equals amount of protein were loaded for each cell type. Results clearly show a higher expression of ABCC2 in HepG2 cells.

Table S1. Size distribution of EVs isolated from cell culture. Described are the cell of origin and the eventual treatment (compound and time) and the particle diameter determined by DLS. Data are presented as mean ± S.D. At least 3 measurements were performed.

Cell of origin (and treatment)	Group	Particle diameter (nm)
HepG2 (Rifampicin, 48 h)	Control	158.7 ± 20.0
	Rifampicin 5 µM	161.8 ± 22.0
	Rifampicin 10 µM	131.1 ± 7.9
	Rifampicin 20 µM	144.1 ± 7.1
HepG2 (Rifampicin, 72 h)	Control	212.3 ± 20.4
	Rifampicin 5 µM	148.9 ± 24.0
	Rifampicin 10 µM	206.3 ± 27.5
	Rifampicin 20 µM	186.8 ± 14.3
HepG2 (Hypericin, 72 h)	Control	175.1 ± 9.3
	Hypericin 0.01 µM	165.8 ± 16.5
	Hypericin 0.1 µM	166.4 ± 9.8
	Hypericin 0.5 µM	177.6 ± 62.6
MCF7 (Rifampicin, 48 h)	Control	241.7 ± 102.2
	Rifampicin 5 µM	193.2 ± 20.4
	Rifampicin 10 µM	247.8 ± 131.0
	Rifampicin 20 µM	227.5 ± 30.0
MCF7 (Rifampicin, 72 h)	Control	247.4 ± 20.2
	Rifampicin 5 µM	253.1 ± 18.7
	Rifampicin 10 µM	257.3 ± 30.1
	Rifampicin 20 µM	244.3 ± 41.4

Table S2. Regulation of ABCC2 in HepG2 cells.

Compound and incubation time	Concentration	ABCC2 protein expression (fold change of control)
Enzalutamide (96 h)	Control	1.00 ± 0.24
	0.8 µM	1.35 ± 0.34
	4 µM	1.07 ± 0.36
	8 µM	1.02 ± 0.25
Hyperforin (48 h)	Control	1.00 ± 0.05
	0.01 µM	1.18 ± 0.30
	0.1 µM	1.25 ± 0.09
	0.5 µM	0.98 ± 0.23
Mitotane (96 h)	Control	1.00 ± 0.24
	10 µM	1.09 ± 0.34
	20 µM	1.13 ± 0.28
	40 µM	1.03 ± 0.52

Described is the ABCC2 protein expression in HepG2 cells treated with compounds previously reported to modulate ABC transporters. ABCC2 expression was determined by UPLC-MS/MS as described in section 4.9 of the manuscript. n = 3-4. No significant differences were observed.

Table S3. Regulation of ABCG2 transporters in MCF7 cells.

Compound and incubation time	Concentration	ABCG2 protein expression (fold change of control)
Enzalutamide (96 h)	Control	1.00 ± 0.18
	0.8 µM	1.21 ± 0.11
	4 µM	1.36 ± 0.39
	8 µM	0.95 ± 0.19
Hyperforin (48 h)	Control	1.00 ± 0.27
	0.01 µM	1.37 ± 0.31
	0.1 µM	1.06 ± 0.02
	0.5 µM	1.02 ± 0.30
Hypericin (72 h)	Control	1.00 ± 0.08
	0.01 µM	0.91 ± 0.20
	0.1 µM	1.21 ± 0.50
	0.5 µM	0.67 ± 0.11
Mitotane (96 h)	Control	1.00 ± 0.18
	10 µM	1.19 ± 0.15
	20 µM	1.05 ± 0.05
	40 µM	0.86 ± 0.49

Described is the ABCG2 protein expression in MCF7 cells treated with compounds previously reported to modulate ABC transporters. ABCG2 expression was determined by UPLC-MS/MS as described in section 4.9 of the manuscript. n = 4. No significant differences were observed.
